# Association between classic and specific bioimpedance vector analysis and sarcopenia in older adults: a cross-sectional study

**DOI:** 10.1186/s13102-022-00559-2

**Published:** 2022-09-14

**Authors:** Ana Claudia Rossini-Venturini, Pedro Pugliesi Abdalla, Priscila Giacomo Fassini, André Pereira dos Santos, Márcio Fernando Tasinafo Junior, Thiago Cândido Alves, Euripedes Barsanulfo Gonçalves Gomide, Tatiane Lopes de Pontes, Karina Pfrimer, Eduardo Ferriolli, Jorge Mota, Maria Reyes Beltran-Valls, Dalmo Roberto Lopes Machado

**Affiliations:** 1 College of Nursing at Ribeirão Preto, University of São Paulo, Avenue of Bandeirantes no 3900, University Campus - Monte Alegre, Ribeirão Preto, SP 14040-902 Brazil; 2 Study and Research Group in Anthropometry, Training and Sport (GEPEATE), São Paulo, Brazil; 3 Ribeirão Preto Medical School, University of São Paulo, Ribeirão Preto, Brazil; 4 School of Physical Education and Sport of Ribeirão Preto, University of São Paulo, Ribeirão Preto, Brazil; 5 Claretiano – University Center, São Paulo, Brazil; 6 Department of Nutrition, University of Ribeirão Preto, Ribeirão Preto, Brazil; 7 The Research Centre in Physical Activity, Health and Leisure (CIAFEL), Faculty of Sports (FADE), Porto, Portugal; 8 LIFE Research Group, University Jaume I, Castellón de la Plana, Spain

**Keywords:** Bioelectrical impedance analysis, Phase angle, Sarcopenia, Older adults, Body composition

## Abstract

**Background:**

To verify (1) the association between classic and *specific* bioelectrical impedance vector analysis (BIVA) with body composition, hydration, and physical performance in older adults with and without sarcopenia; (2) which BIVA most accurately distinguishes sarcopenia.

**Methods:**

A sample of 94 older adults with and without sarcopenia (29 men and 65 women, 60–85 years) was evaluated. The classic and *specific* BIVA procedures, Dual energy X-ray absorptiometry (DXA), and deuterium dilution were performed. Sarcopenia was defined by muscle weakness and low skeletal muscle index, while severity was indicated by low physical performance.

**Results:**

The BIVA's potential to monitor hydration and muscle mass loss in older adults seems feasible. Classic and *specific* BIVA were able to distinguish sarcopenia in women (*p* < 0.001), but not in men. When the sarcopenia criteria were individually analyzed, both classic and *specific* BIVA were able to distinguish low skeletal muscle index in women, while only classic BIVA did for men. For the criterion of slow physical performance, only the classic BIVA showed severity differences for women. The vectors of adults without sarcopenia of both sexes tended to be positioned in the left region of the ellipses, revealing a predominance of soft tissues.

**Conclusions:**

Classic BIVA has a distinct sarcopenic association with body composition, hydration, and physical performance in older adults, while *specific* BIVA was similar between groups. Both BIVAs are sensible to detect female morphological changes (skeletal muscle index) but not for functional (handgrip, 6-min walk test) sarcopenia criteria. These procedures are promising tools for monitoring sarcopenia risks during aging.

## Background

Sarcopenia is a progressive and generalized skeletal muscle illness [1] that is related to low strength [2] and muscle quantity [1] that is related to increased adverse consequences including falls, fractures, physical disability and mortality [1, 3]. Moreover, it has a strong impact on the ability to perform activities of daily living [4] and greatly reduces the quality of life [5]. The diagnosis of sarcopenia is established by the presence of low muscle strength and muscle quantity [3], and is considered severe when low physical performance is detected [3]. Usually, during ageing, bone mineral content (BMC), lean soft tissue (LST) and total body water (TBW) decrease throughout life, while the fat mass (FM) increases and is redistributed to the abdominal region [6, 7]. Thus, analyzing and monitoring hydration and body composition (BC) changes during ageing is necessary. Since LST major component is water (about 76%) and that it is commonly reduced during ageing, TBW losses may affect muscle function [8, 9].

Several techniques are available for BC analysis, with their advantages and disadvantages [10]. Although Dual-energy X-ray absorptiometry (DXA) is a reference method for BMC, also consists in a three-compartment model [11] for BC, being recognized as a precise and accurate technique for determining FM, LST and BMC [12]. The hydration can be accurately assessed through dilution techniques using deuterium [13], as an important measure for a healthy body [9]. However, DXA and dilution techniques are expensive, need specialized technicians to analyze the exams, are difficult to use in the field setting [14, 15], and are available in a few research centers. In this way, simple methods proposed to monitor TBW and BC are desired during ageing. Bioelectrical impedance analysis (BIA) is a non-invasive, low-cost, easily applicable method, and can be an alternative to TBW and BC diagnostic tool for routine examinations in clinical and research practice [16, 17]. Nevertheless, BIA has limitations in terms of development of *specific* equations [18] that is why bioelectrical impedance vector analysis (BIVA) has been used.

BIVA is based on the analysis of impedance vectors, designed on an RXc-score graph to reference values (tolerance ellipses) or for intergroup comparisons—confidence ellipses [18]. In the classic BIVA analyses, bioelectrical values are standardized for the subject's height (R/H: resistance standardized for height; Xc/H: reactance standardized for height) to remove the effect of conductor length [19, 20]. Instead, the *specific* BIVA corrects the bioelectrical values for height and transverse areas (Rsp: resistance standardized for height and transverse areas; Xcsp: reactance standardized for height and transverse areas), to reduce the effect of body dimensions [21, 22]. According to the classic BIVA [19], alterations of bioelectrical vectors along the major axis of tolerance ellipses show changes in TBW (fluid excess in the direction of the lower pole, dehydration towards the upper pole). In relation to *specific* BIVA [23], the major axis refers to variations in FM% (higher values towards the upper pole). The minor axis refers to the variations in body cell mass, skeletal muscle mass in particular, and ICW/ECW (intra cellular water/extracellular water) ratio (higher values on the left side).

Since sarcopenia represents muscle mass reduction, BIVA could be used to identify these muscle changes. However, which BIVA best defines sarcopenia, or how bioelectrical impedance vectors are associated with BC, hydration, and diagnostic criteria for sarcopenia in older adults have not been established up until now. Thus, our objectives were: (1) To evaluate the association between classic and *specific* BIVA with BC, hydration, and physical performance of older adults with and without sarcopenia; (2) To verify which BIVA (classic or *specific*) is more accurate for distinguishing sarcopenia for both sexes. We hypothesize that since the *specific* BIVA corrects the BIA values by transverse areas, it better distinguishes sarcopenia for both sexes than classic BIVA.

## Method

### Design and study population

In this study, we adopted a cross-sectional design to evaluate physically independent community-dwelling older adults, living in a city in southeastern Brazil. The study was conducted from October 2016 to May 2017. This manuscript followed the guidelines from The Strengthening the Reporting of Observational Studies in Epidemiology (STROBE) conference list, and the completed checklist is attached.

A sample of older adults aged between 60 and 85 years old of both sexes were considered for analysis. They were recruited in special projects for seniors (Exercise Program for older adults on campus on the interior of the University of São Paulo) and health services in a great regional community from a mid-west zone of Brazil. Former research participants (Fragility project) at the university were also invited to participate in the study. The approach was made by phone and personal invitation. The inclusion criteria were: adults aged 60–85 years old, both sexes, who walked independently. The exclusion criteria were: the presence of diseases that restrict mobility or muscle strength; presence of unstable cardiovascular condition; acute infection; back pain; prostheses, individuals with a diagnosis of cancer or uncontrolled diseases, who presented sequel of stroke, experienced a weight loss more than 3 kg (kg) in the last 3 months, had a cognitive limitation that restricts understanding and taking tests, who did not complete all the stages, or desired to withdraw from the study.

To the sample size calculation was considered the desired maximum error (ε) and degree of confidence (Zy), previously knowing the population variability (σ^2^) [24]. For this, we used the variable with the greatest variability (FM; SD = 8.7 kg) expected for such a population [25]. Once the predetermined error estimate (ε ≤ 1.8 kg) and maximum desired error (5%) the ideal n for the study [24] was defined (n = 90). All participants volunteered, received an explanation about the study's objectives and signed the written consent in agreement with the Declaration of Helsinki. The study was approved by the Ethical Review Board of Hospital das Clinicas at the Medical School of University of Sao Paulo (HC-FMRP/USP).

### Procedures

A multidisciplinary health team (nurses, nutritionists, pharmacists, physical education professors, physicians, and physiotherapists) performed data collection. All procedures, for each participant, were completed during one visit to the laboratories at the Hospital das Clinicas at the Medical School of University of Sao Paulo at Ribeirao Preto (HCFMRP/USP). Subjects came to the laboratory after an overnight fast (8 h fast), abstaining from vigorous exercises, no caffeine and alcohol during the preceding 24 h.

### Cognition assessment

The cognition was assessed using the short version of the Mini-mental state examination (MMSE), which presents a maximum score of 19 points [26]; the individuals with scored ≤ 12 were considered with cognitive limitations.

### Anthropometry

Weight (kg) and height (cm) were measured according to standardized procedures [27]. Body mass index (BMI) was derived (kg/m^2^). Upper arms, waist, and calf (cm) circumferences were measured by an anthropometric tape.

### Dual-energy X-ray absorptiometry

BC was determined by DXA (Hologic® scanner, model QDR4500W; version 11.2, Bedford, MA). Skeletal muscle mass index (SMI) was derived from Appendicular lean soft tissue (ALST) and squared height ratio (kg/m^2^). The calibration and measurements were following the manufacturer's instructions and were always carried out by the same technician. The examination was performed according to standardized procedures previously informed [28, 29]. The DXA measurements included absolute and relative values (ALST and ALST%; FM and FM%; BMC and BMC%).

### Bioelectrical impedance

The bioelectrical impedance measurements were performed with BIA Imp DF50 Body Composition Analysis (ImpediMed, Brisbane, Queensland, Australia) according to international standard criteria [17]. The participants were in the supine position with a leg opening of 45° compared to the median line of the body and the upper limbs and with a distance of 30° from the trunk. The skin was cleaned with alcohol, then two electrodes were placed on the right hand back and two electrodes on the neck of the corresponding foot [30]. Phase angle (degrees) was calculated as the arctangent of Xc/H * 180°/π.

Bioelectrical impedance vector analysis was carried out using the classic and *specific* BIVA methods. The classic BIVA was applied, adjusting individual vectors for height (H) in meters (R/H, Ohm/m; Xc/H Ohm/m) [19] to eliminate conductor length effect. The characteristics of the sample were compared with the concentric tolerance ellipses (50%, 75% and 95% of cases) representing the variability of the reference population [31]. The *specific* BIVA was applied to compensate for the whole effect of conductor volume. The bioelectrical values were multiplied by a correction factor (A/L, in centimeters; R*A/L, Ohm/cm; Xc*A/L, Ohm/cm). Where L = 1.1*H (cm) and A is estimated cross-sectional area:

$${\text{A}} = \left( {0.45*{\text{arm area}} + 0.10*{\text{waist area}} + 0.45*{\text{calf area}}} \right)\;\left( {{\text{in cm}}^{2} } \right),$$

Note that segment area = C^2^/4π, and C is the circumference of the arm, waist, and calf in centimeters [23, 32]

The coefficients were assigned considering the different contribution of body segments to resistance and the proportions of total body length. Italian older adults’ bioelectrical values [33] were used as reference.

### Sarcopenia identification

The criteria established by the EWGSOP [3] to identify sarcopenia were: decreased levels of handgrip strength and SMI. Physical performance (6-min walk test) was tested to check the severity of sarcopenia. As described below, the cutoff points for muscle strength, muscle quality and physical performance were those suggested by EWGSOP [34]. This choice is justified considering that they were better to define sarcopenia in the Brazilian population [35].

### Muscle strength

The handgrip strength was measured (kg) using a dynamometer Jamar®, model 5030 J1, and the protocol followed the American Society of Hand Therapists recommendations [36]. The participants were verbally stimulated and made three attempts with their dominant hands, with 1-min rest between attempts. The highest measure value was recorded [35, 37]. Muscle strength was considered low when handgrip strength was below 30 kg or 20 kg for men and women, respectively [38].

### Muscle quantity

SMI was the criterion used for muscle mass, where ALST is divided by squared height (kg/m^2^). The SMI below 7.26 kg/m^2^ and 5.45 kg/m^2^ was considered low for men and women, respectively [39].

### Physical performance

To assess physical performance the 6-min walk test (6MWT) was performed on a flat, non-slip surface, in a space of 30-m, with calibrated markings every 3 m. Before the test, the participants were asked to walk as fast as possible for 6 min. Then, after a verbal command, they began to walk. Although time was not paused during execution, the participants could slow down or stop to rest and return to the test at will. The total walked distance was noted in meters. Sarcopenia was considered severe when 6MWT ≤ 400 m [40]. Participants with sarcopenia who had 6MWT < 400 m had the disease classified as severe [3]

### Total body water

TBW was assessed by isotopic dilution of deuterium oxide. This method is based on stable isotopes and consists of ingesting a deuterium oxide dose and determining, by mass spectrometry, deuterium enrichment in a sample of body water (e.g., saliva). Due to the difference in enrichment before and after ingestion of the dose, the TBW is precisely determined [13]. Each participant had an 8-h overnight fast. Afterwards, a fixed dose of 70 ml of 7% deuterium oxide was consumed, followed by 50 ml of water to rinse the mouth, this last process was repeated to ensure that there was no water left in the bottle. Saliva samples of each participant were collected before ingesting the deuterium oxide dose (basal) and 3 h later. Deuterium enrichment was determined by isotopic ratio mass spectrometry (IRMS Hydra, Europa Scientific, Cheshire, United Kingdom).

### Statistical analysis

Descriptive statistics using measures of central tendency were used to describe the characteristics of the sample. To verify the normality of the data, the Shapiro–Wilk test was applied. Comparisons between men and women and between individuals with and without sarcopenia were performed using Student’s t-test for independent samples for parametric data and the Mann–Whitney U test for nonparametric data. The association between body composition, hydration, physical performance, and bioelectrical variables was investigated using Pearson’s correlation analysis. The Hotelling's t-squared statistic (t^2^) was used to compare the impedance vectors mean between sarcopenic and non-sarcopenic groups. Statistical significance was pre-determined as *p* < 0.05. SPSS 23.0 and NCSS 2020 were used for all statistical calculations.

## Results

Table 1 shows the characteristics of the eligible participants. From the EWGSOP [3] criteria, sarcopenia was found in four men (13.8% of the male sample) and six women (9.2% of the female sample). Sarcopenic older adults showed statistically lower calf circumference, ALST, SMI, and handgrip strength than those older adults without sarcopenia (*p* < 0.05). Sarcopenic women were older and had higher R, Z, R/H, and Z/H values, while women without sarcopenia were heavier and had higher upper arm circumference, BMI, PA, Xcsp, and FM values. Table 1 also shows anthropometric, body composition, hydration, bioelectrical and physical performance variables, which have significant differences between sexes (*p < 0.05). Men without sarcopenia were taller, heavier, had higher values of waist circumference, BMC (kg and %), ALST (kg and %), SMI, TBW (liter and %), grip strength, and lower values of FM (kg and %) and for all bioelectrical variables (except the PA) compared to women without sarcopenia. And sarcopenic men showed higher values of ALST (kg and %), SMI, TBW%, handgrip strength, and lower R, Z, R/H, and Z/H than sarcopenic women.

**Table 1:** Descriptive values of older adults, including the correlation between bioelectrical variables and difference test

	Men					Women				
	Without sarcopenia (n = 25)		With sarcopenia (n = 04)		*p*	Without sarcopenia (n = 59)		With sarcopenia (n = 06)		*p*
	Mean (SD)	CI-95%	Mean (SD)	CI-95%		Mean (SD)	CI-95%	Mean (SD)	CI-95%	
Age (years)	71.0 (7.6)	67.8–74.1	76.8 (6.6)	66.2–87.3	0.164	69.5 (5.8)	68.0–71.0	75.3 (5.6)	69.4–81.3	0.023
*Anthropometric variables*										
Height (cm)	169.2 (7.6)	166.1–172.3	164.3 (9.8)	148.6–179.9	0.255	156.4 (6.0)*	154.8–157.9	154.3 (7.4)	146.5–162.0	0.418
Weight (kg)	75.1 (12.8)	69.8–80.4	62.3 (16.7)	35.7–88.8	0.085	67.8 (11.1)*	64.9–70.7	54.4 (10.2)	43.8–65.1	0.014
Upper arm crf (cm)	29.4 (3.3)	28.0–30.7	26.1 (4.4)	19.1–33.1	0.090	30.3 (3.7)	29.3–31.2	26.5 (3.7)	22.5–30.3	0.017
Waist crf (cm)	92.9 (10.6)	88.5–97.3	89.4 (16.9)	62.5–116.2	0.576	86.9 (10.0)*	84.3–89.5	79.6 (11.0)	68.0–91.2	0.096
Calf crf (cm)	36.4 (3.1)	35.1–37.7	31.2 (3.2)	26.2–36.3	0.005	35.3 (2.9)	34.5–36.0	31.7 (2.3)	29.2–34.1	0.004
BMI (kg/m^2^)	26.2 (3.5)	24.7–27.6	22.8 (4.5)	15.7–30.0	0.102	27.7 (4.3)	26.6–28.8	22.8 (3.5)	19.1–26.5	0.009
*Body composition variables*										
FM (kg)	22.2 (6.6)	19.5–24.9	19.6 (10.6)	2.6–36.5	0.661	28.2 (6.9)*	26.4–30.0	22.1 (7.4)	14.4–29.9	0.046
FM (%)	29.2 (6.5)	26.5–31.0	29.7 (9.4)	14.7–44.7	0.900	41.2 (5.2)*	39.8–42.6	39.8 (6.7)	32.7–46.9	0.548
BMC (kg)	2.6 (0.5)	2.4–2.8	1.8 (0.4)	1.2–2.4	0.079	1.9 (0.3)*	1.8–2.0	1.8 (0.3)	1.4–2.1	0.287
BMC (%)	3.5 (0.5)	3.3–3.7	3.0 (0.7)	1.9–4.1	0.123	2.9 (0.5)*	2.8–3.0	3.3 (0.7)	2.6–4.1	0.144
ALST (kg)	21.5 (4.0)	19.9–23.2	16.8 (2.9)	12.1–21.4	0.032	15.0 (2.5)*	14.3–15.6	11.5 (1.4)*	10.0–12.9	0.001
ALST (%)	29.3 (3.5)	27.8–30.7	27.9 (3.8)	21.9–34.0	0.370	22.5 (2.4)*	21.9–23.2	21.6 (2.4)*	19.1–24.1	0.345
SMI (kg/m^2^)	7.9 (1.0)	7.5–8.3	6.5 (0.5)	5.6–7.3	0.009	6.5 (1.0)*	6.2–6.7	5.1 (0.3)*	4.8–5.4	0.001
*Hydration variables*										
TBW (l)	40.5 (6.1)	36.8–44.2	32.0 (7.5)	13.3–50.7	0.055	28.8 (3.4)*	27.5–30.1	25.3 (3.0)	17.8–32.7	0.165
TBW (%)	53.7 (5.8)	50.2–57.2	55.9 (4.2)	45.5–66.3	0.549	44.0 (3.7)*	42.6–45.5	43.5 (2.4)*	37.4–49.6	0.815
*Bioelectrical variables*										
R (ohm)	475.1 (64.6)	448.4–501.8	511.6 (56.1)	422.3–600.1	0.297	541.1 (68.9)*	523.2–559.1	638.0 (84.9)*	548.9–727.1	0.002
Xc (ohm)	48.9 (12.9)	43.5–54.2	51.0 (11.7)	32.4–69.6	0.762	56.9 (10.4)*	54.2–59.6	54.5 (21.8)	31.6–77.3	0.308
Z (ohm)	474.9 (65.9)	447.7–502.1	514.2 (57.0)	423.5–604.8	0.272	545.8 (68.5)*	527.9–563.6	640.5 (86.0)*	550.3–730.7	0.002
PA (°)	6.0 (1.6)	5.3–6.2	5.6 (0.7)	4.4–6.8	0.669	6.0 (1.0)	5.7–6.3	4.7 (1.7)	2.9–6.5	0.043
R/H (ohm/m)	281.1 (38.2)	265.3–296.8	313.1 (46.7)	238.8–387.3	0.141	346.0 (42.0)*	335.1–357.0	413.5 (49.9)*	361.1–465.9	0.004
Xc/H (ohm/m)	29.0 (7.7)	25.8–32.1	31.3 (8.4)	17.9–44.8	0.578	36.4 (6.7)*	34.7–38.2	35.3 (14.1)	20.5–50.1	0.248
Z/H (ohm/m)	280.9 (38.8)	264.9.1–296.9	314.7 (47.3)	239.5–389.9	0.127	349.0 (41.5)*	338.2–359.8	415.1 (50.6)*	362.0–468.2	0.004
Rsp (ohm*cm)	372.6 (43.5)	354.6–390.5	356.7 (109.5)	182.4–531.0	0.793	433.4 (71.3)*	414.8–452.0	422.6 (110.9)	306.2–539.1	0.739
Xcsp (ohm*cm)	39.1 (12.1)	34.1–44.1	35.7 (13.5)	14.3–57.2	0.612	45.7 (9.8)*	43.2–48.3	35.3 (15.9)	18.7–52.0	0.024
Zsp (ohm*cm)	374.8 (43.9)	356.6–392.9	358.5 (110.3)	183.1–534.0	0.789	435.9 (71.6)*	417.2–454.5	424.3 (111.4)	307.3–541.2	0.720
*Physical performance variables*										
6MWT (m)	452.4 (106.9)	408.3–496.5	465.9 (49.2)	387.7–544.1	0.150	407.0 (97.2)	381.6–432.3	440.9 (66.5)	371.2–510.7	0.202
Hand grip Strenght (kg)	37.1 (8.3)	33.7–40.5	28.3 (2.1)	25.0–31.5	0.045	24.5 (4.4)*	23.3–25.6	18.7 (1.2)*	17.4–19.9	0.001
*Correlations*										
r R-Xc	0.017		0.988			0.465		0.907		
r R/H-Xc/H	0.075		0.998			0.447		0.944		
r Rsp-Xcsp	0.489		0.967			0.659		0.742		

crf: circumference; BMI: body mass index; R: resistance; Xc: reactance; Z: vector length; PA: phase angle; R/H: resistance standardized for height; Xc/H: reactance standardized for height; Z/H: vector length standardized for height; Rsp: resistance standardized for height and transverse areas; Xcsp: reactance standardized for height and transverse areas; Zsp: vector length standardized for height and transverse areas; 6MWT: 6-min walk test; ALST: Appendicular lean soft tissue; FM: fat mass; BMC: bone mineral content; TBW: total body water; SMI: Skeletal muscle mass index; r R-Xc: correlation between R and Xc; r R/H-Xc/H: correlation between R/H and Xc/H; r Rsp-Xcsp: correlation between Rsp and Xcsp

*Sex differences (*p* < 0.05); Independent t test for parametric

^U^Mann–Whitney U test for non-parametric data

The highest correlation values for classic (R/H-Xc/H) and *specific* (Rsp-Xcsp) bioelectric variables were observed in sarcopenic groups, both in men and women. Complete correlation analysis between body composition, hydration, physical performance and bioelectrical variables, considering sexes and sarcopenic status were shown in Tables 2 and 3.

**Table 2:** Correlation between body composition, hydration, physical performance and bioelectrical variables in men without/with sarcopenia

	BIA			Classic BIVA			Specific BIVA			PA
	R	Xc	Z	R/H	Xc/H	Z/H	Rsp	Xcsp	Zsp	
*Men without sarcopenia*										
FM (kg)	− 0.459*	0.074	− 0.431*	− 0.490*	0.055	− 0.463*	0.745**	0.490*	0.747**	0.255
FM%	− 0.298	0.094	− 0.287	− 0.208	0.133	− 0.199	0.576**	0.372	0.576**	0.184
BMC (kg)	− 0.473*	− 0.202	− 0.460*	− 0.640**	− 0.279	− 0.628**	0.290	0.138	0.290	0.049
BMC%	− 0.032	− 0.317	− 0.055	− 0.056	− 0.319	− 0.080	− 0.447*	− 0.389	− 0.450*	− 0.259
ALST (kg)	− 0.321	0.041	− 0.310	− 0.554**	− 0.072	− 0.543**	0.384	0.332	0.390	0.230
ALST%	0.306	0.031	0.273	0.198	− 0.017	0.167	− 0.474*	− 0.217	− 0.471*	− 0.055
SMI (kg/m^2^)	− 0.565**	0.105	− 0.564**	− 0.680**	0.048	− 0.681**	0.317	0.469*	0.327	0.409*
TBW (l)	− 0.693**	− 0.218	− 0.647*	− 0.849**	− 0.357	− 0.813**	0.323	0.340	0.327	0.304
TBW%	0.072	− 0.170	0.028	0.164	− 0.109	0.122	− 0.922**	− 0.579*	− 0.920**	− 0,190
6MWT (min)	− 0.060	0.430*	− 0.121	− 0.209	0.351	− 0.270	0.141	0.477*	0.154	0.472*
Hand grip Strenght (kg)	− 0.012	0.283	− 0.021	− 0.191	0.190	− 0.199	− 0.131	0.217	− 0.122	0.304
*Men with sarcopenia*										
FM (kg)	0.410	0.273	0.407	0.027	0.068	0.027	0.924	0.796	0.923	0.199
FM%	0.548	0.417	0.545	0.186	0.224	0.186	0.960*	0.864	0.959*	0,347
BMC (kg)	− 0.195	− 0.226	− 0.196	− 0.406	− 0.349	− 0.405	0.318	0.240	0.317	− 0.253
BMC%	− 0.494	− 0.367	− 0.492	− 0.232	− 0.236	− 0.232	− 0.717	− 0.620	− 0.716	− 0.308
ALST (kg)	− 0.229	− 0.362	− 0.232	− 0.588	− 0.549	− 0.588	0.522	0.300	0.519	− 0.429
ALST%	− 0.675	− 0.559	− 0.673	− 0.338	− 0.377	− 0.339	− 0.990*	− 0.935	− 0.990*	− 0.495
SMI (kg/m^2^)	0.128	0.018	0.125	− 0.240	− 0.183	− 0.240	0.762	0.627	0.761	− 0.046
TBW (l)	− 0.361	− 0.528	− 0.365	− 0.674	− 0.670	− 0.675	0.629	0.293	0.626	− 0.596
TBW%	− 0.462	− 0.290	− 0.458	− 0.109	− 0.116	− 0.109	− 0.999*	− 0.912	− 0.999*	− 0.209
6MWT (min)	− 0.364	− 0.384	− 0.365	− 0.534	− 0.484	− 0.533	0.147	0.058	0.146	− 0.404
Hand grip Strenght (kg)	− 0.813	− 0.893	− 0.815	− 0.960*	− 0.959*	− 0.960*	− 0.208	− 0.447	− 0.211	− 0.924

crf: circumference; BMI: body mass index; R: resistance; Xc: reactance; Z: vector length; PA: phase angle; R/H: resistance standardized for height; Xc/H: reactance standardized for height; Z/H: vector length standardized for height; Rsp: resistance standardized for height and transverse areas; Xcsp: reactance standardized for height and transverse areas; Zsp: vector length standardized for height and transverse areas; ALST: Appendicular lean soft tissue; FM: fat mass; BMC: bone mineral content; TBW: total body water; SMI: Skeletal muscle mass index; %: relative values (ratio) to body weight; 6MWT: 6-min walk test; r R-Xc: correlation between R and Xc; r R/H-Xc/H: correlation between R/H and Xc/H; r Rsp-Xcsp: correlation between Rsp and Xcsp

*p < 0.05; **p < 0.001

**Table 3:** Correlation between body composition, hydration, physical performance and bioelectrical variables in women without/with sarcopenia

	BIA			Classic BIVA			Specific BIVA			PA
	R	Xc	Z	R/H	Xc/H	Z/H	Rsp	Xcsp	Zsp	
*Women without sarcopenia*										
FM (kg)	− 0.257*	− 0.176	− 0.272*	− 0.339**	− 0.221	− 0.358**	0.638**	0.452**	0.638**	0.007
FM%	− 0.097	− 0.096	− 0.102	− 0.104	− 0.099	− 0.109	0.399**	0.249	0.398**	− 0.049
BMC (kg)	− 0.175	− 0.067	− 0.184	− 0.314*	− 0.156	− 0.327*	0.242	0.230	0.243	0.064
BMC%	0.112	0.030	0.120	0.086	0.008	0.094	− 0.531**	− 0.397**	− 0.532**	− 0.049
ALST (kg)	− 0.312*	− 0.068	− 0.324*	− 0.425**	− 0.134	− 0.442**	0.500**	0.474**	0.502**	0.176
ALST%	0.009	0.134	0.001	− 0.009	0.133	− 0.001	− 0.313*	− 0.112	− 0.312*	0.157
SMI (kg/m^2^)	− 0.474**	− 0.125	− 0.496**	− 0.452**	− 0.095	− 0.477**	0.457**	0.495**	0.460**	0.244
TBW (l)	− 0.063	− 0.098	− 0.107	− 0.201	− 0.188	− 0.251	0.579**	0.381*	0.579**	− 0.038
TBW%	0.035	0.203	0.021	0.098	0.237	0.085	− 0.388*	− 0.118	− 0.386*	0.254
6MWT (min)	0.202	0.330*	0.195	0.138	0.279*	0.132	− 0.249	− 0.027	− 0.247	0.202
Hand grip Strenght (kg)	− 0.017	− 0.048	− 0.031	− 0.129	− 0.126	− 0.145	0.188	0.137	0.188	− 0.037
*Women with sarcopenia*										
FM (kg)	− 0.261	− 0.554	− 0.267	− 0.429	− 0.608	− 0.433	0.627	− 0.035	0.623	− 0.634
FM%	− 0.347	− 0.557	− 0.352	− 0.400	− 0.569	− 0.404	0.618	− 0.020	0.614	− 0.598
BMC (kg)	0.102	− 0.137	0.099	− 0.194	− 0.240	− 0.196	− 0.045	− 0.199	− 0.046	− 0.260
BMC%	0.190	0.331	0.194	0.184	0.329	0.188	− 0.565	− 0.131	− 0.562	0.334
ALST (kg)	0.343	− 0.054	0.337	0.011	− 0.182	0.006	0.540	0.207	0.538	− 0.213
ALST%	0.504	0.688	0.508	0.611	0.713	0.614	− 0.430	0.215	− 0.426	0.715
SMI (kg/m^2^)	− 0.078	− 0.462	− 0.086	− 0.207	− 0.513	− 0.214	0.542	− 0.044	0.538	− 0.573
TBW (l)	− 0.158	− 0.359	− 0.160	− 0.389	− 0.438	− 0.389	0.334	− 0.089	0.331	− 0.464
TBW%	0.276	0.469	0.278	0.497	0.543	0.498	− 0.218	0.208	− 0.215	0.568
6MWT (min)	0.393	0.433	0.394	0.325	0.398	0.326	− 0.477	− 0.008	− 0.475	0.417
Hand grip Strenght (kg)	− 0.107	0.244	− 0.100	0.006	0.290	0.012	− 0.560	− 0.100	− 0.558	0.370

crf: circumference; BMI: body mass index; R: resistance; Xc: reactance; Z: vector length; PA: phase angle; R/H: resistance standardized for height; Xc/H: reactance standardized for height; Z/H: vector length standardized for height; Rsp: resistance standardized for height and transverse areas; Xcsp: reactance standardized for height and transverse areas; Zsp: vector length standardized for height and transverse areas; ALST: Appendicular lean soft tissue; FM: fat mass; BMC: bone mineral content; TBW: total body water; SMI: Skeletal muscle mass index; 6MWT: 6-min walk test; r R-Xc: correlation between R and Xc; r R/H-Xc/H: correlation between R/H and Xc/H; r Rsp-Xcsp: correlation between Rsp and Xcsp

*p < 0.05; **p < 0.001

Table 2 emerge that specific vector length (Zsp) is significantly associated with %FM in non-sarcopenic and sarcopenic individuals, thus suggesting that specific BIVA could be helpful to assess sarcopenic obesity diagnosis.

**Fig. 1:**
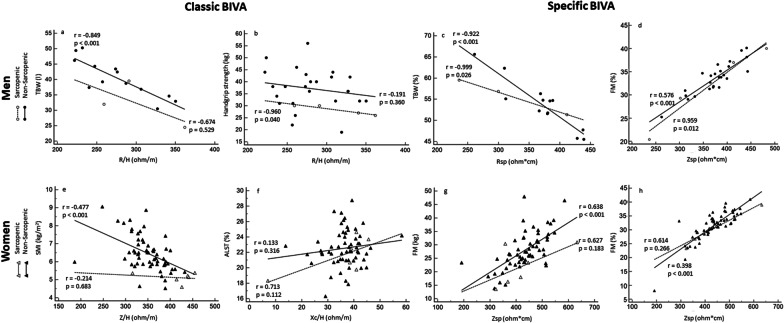
Bivariate correlations between classic and *specific* BIVA with body composition and handgrip for both sexes. *TBW* total body water, *SMI* skeletal muscle index, *ALST* appendicular lean soft tissue, *FM* fat mass, *R/H* resistance standardized for height, *Rsp* resistance standardized for height and transverse areas, *Z/H* vector length standardized for height, *Xc/H* reactance standardized for height, *Zsp* vector length standardized for height and transverse areas

Figure 1 graphically shows the greatest correlations found (Tables 2 and 3) between bioelectrical impedance variables (Classic and *Specific* BIVA) and hydration (TBW, TBW%), physical performance (Handgrip strength) and body composition (SMI, ALST, FM) variables of older adults with and without sarcopenia. The classic BIVA of men without sarcopenia showed inverse significantly high correlation between R/H (r = − 0.849) and TBW, as seen in Fig. 1a. For sarcopenic men, Handgrip strength showed a negative and significant very high correlation with R/H (r = − 0.960) (Fig. 1b).

In the *specific* BIVA, the sarcopenic men showed significantly quasi-perfect correlation (r = − 0.922) between Rsp and TBW% (Fig. 1c). Likely, in men without sarcopenia, the TBW% also showed a higher association (r = 0.922) with BIVA parameters (Rsp; Fig. 1c).

For the women parameters, Classic BIVA of women without sarcopenia shown the highest correlation (r = − 0.477) occurred between Z/H and SMI (Fig. 1e). Again, the sarcopenic women, showed a non-significant, but positive and high correlation between ALST% and Xc/H (r = 0.713), as seen in Fig. 1f.

In the *specific* BIVA, the women without sarcopenia presented moderated (r = 0.638) and positively significant correlation between Zsp and FM. Similarly, in sarcopenic women, the FM also indicated positive, but non-significant moderate correlation (r = 0.623) with *specific* bioelectrical variables (Zsp, Fig. 1g). Furthermore, Tables 2 and 3 emerge that Zsp was significantly associated with FM% for men and women without and with sarcopenia, like Fig. 1d, h.

**Fig. 2:**
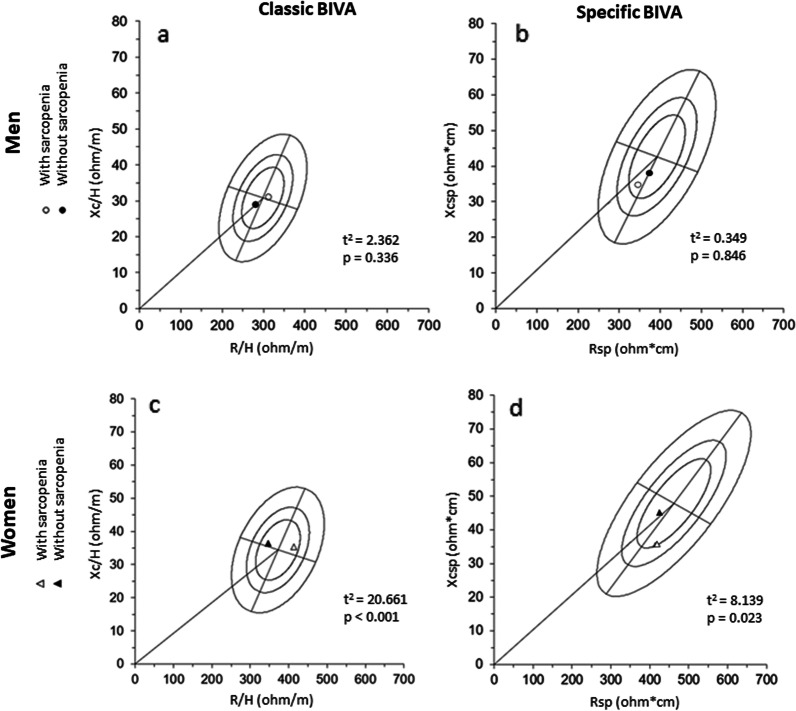
Classic and *specific* mean vectors of older adults with and without sarcopenia. Filled circles: men without sarcopenia; Hollow circles: men with sarcopenia; Filled triangles: women without sarcopenia; Hollow triangles: women with sarcopenia; **a** men classic BIVA; **b** men *specific* BIVA; **c** women classic BIVA; **d** men *specific* BIVA. *R/H* resistance standardized for height, *Xc/H* reactance standardized for height, *Rsp* resistance standardized for height and transverse areas, *Xcsp* reactance standardized for height and transverse areas, t^2^: Hotelling's t-squared statistic

The BIVA accuracy for distinguishing sarcopenia is shown in Fig. 2, as classic and *specific* BIVA of older adults by sexes and sarcopenia status.

The classic BIVA vectors of men groups with and without sarcopenia were not statistically different (T^2^ = 2.362; *p* = 0.336; Fig. 2a). But women are (T^2^ = 20.661; *p* < 0.001; Fig. 2c), demonstrating the ability of classic BIVA to discriminate sarcopenia in women due to higher values of R/H, Z/H, and the lower PA of women with sarcopenia As noted, the Classic BIVA mean vectors of sarcopenia groups for both sexes were located within the 50% tolerance ellipses (central circle), like the healthy reference population, indicating relatively normal tissue impedance properties. Furthermore, the mean vector for men without and with sarcopenia fell into the third and second quadrant, while for women, the mean vectors fell into the first and second quadrant, respectively. As expected, the classic mean vectors of men and women without sarcopenia (filled figures) were positioned in the left side of tolerance ellipses, suggesting higher values of body cell mass (Fig. 2a, c). In addition, the vector of men without sarcopenia was in the lower pole, demonstrating more TBW, while for men with sarcopenia (hollow circle), the vector mean was situated in the higher pole, representing less TBW (Fig. 2a). For women, both sarcopenia groups are at the upper pole, demonstrating no differences in their TBW. Thus, in terms of vector length, the classic BIVA (Z/H) is indicative of TBW).

In *specific* BIVA (like the classic BIVA), the mean vectors of the men sarcopenia groups (Fig. 2b) were not significantly different (T^2^ = 0.349; *p* = 0.846), but women are (T^2^ = 8.139, *p* = 0.023, Fig. 2d). For the men's sarcopenia groups mean vectors fell into the third quadrant and were found inside the 50% tolerance ellipses. Their proximity in the graphic ellipses (filled or hollow figures) already suggested no differences between sarcopenia groups, confirmed by the *p* values (*p* > 0.05). Women, in turn (Fig. 2d), showed mean of vectors of sarcopenia groups significantly distinguished (*p* = 0.023). The mean vectors of women groups without and with sarcopenia were located both within the 50% tolerance ellipses, into the third and fourth quadrants, respectively (Fig. 2d).The cases without sarcopenia (filled figures) towards the left side of tolerance ellipses, indicating higher values of body cell mass, skeletal muscle mass in particular, and ICW/ECW ratio, while the cases with sarcopenia fell on the right side, indicating lower values of body cell mass, skeletal muscle mass and ICW/ECW ratio. Furthermore, the vector of men without sarcopenia was towards the upper pole, demonstrating higher values of %FM, while for men with sarcopenia (hollow circle), the vector mean was situated towards the lower pole, representing lower %FM (Fig. 2b). For women, the behavior was the same (Fig. 2d). So, in terms of vector length, the *specific* BIVA is indicative of %FM, variations in body cell mass, skeletal muscle mass and ICW/ECW.

**Fig. 3:**
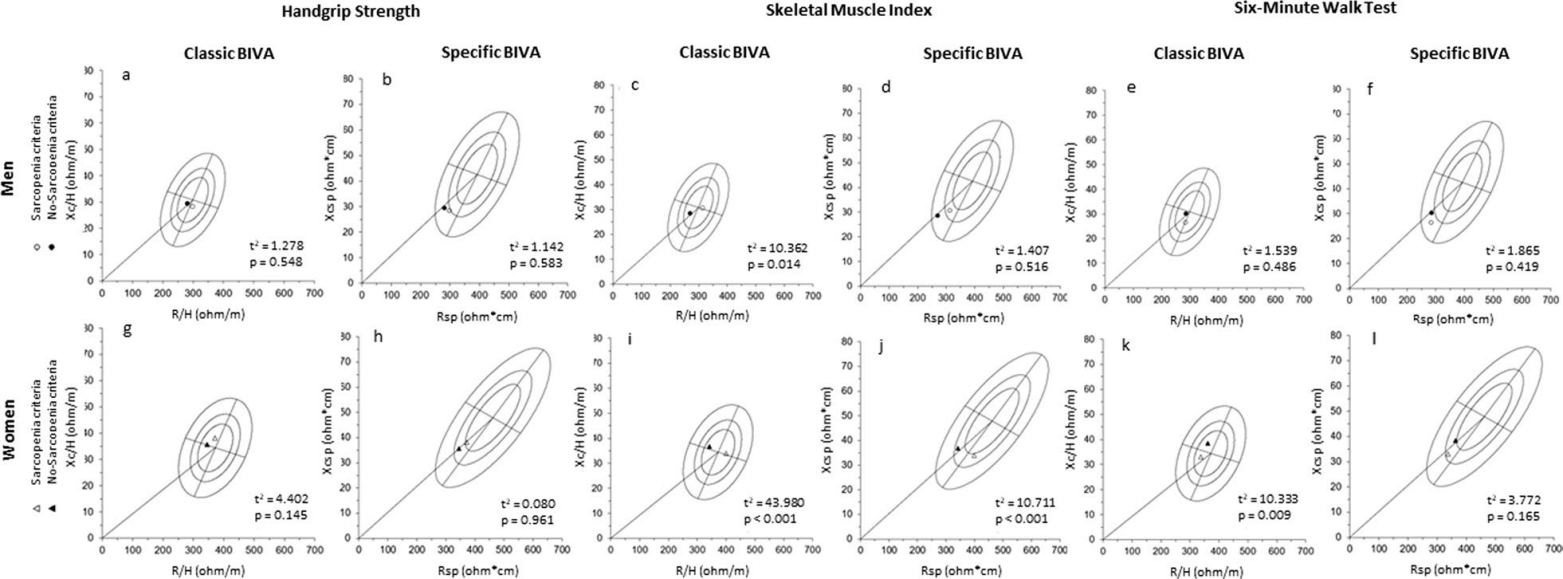
Classic and *specific* BIVA of older adults with low/normal values of the sarcopenia criteria. Filled circles: men with values above the cutoff point; Hollow circles: men with values below the cutoff point; Filled triangles: women with values above the cutoff point; Hollow triangles: women with values below the cutoff point. **a** men with normal-low strength and classic BIVA; **b** men with normal-low strength and *specific* BIVA; **c** men with normal-low muscle quantity and classic BIVA; **d** men with normal-low muscle quantity and *specific* BIVA; **e** men with normal-low physical performance and classic BIVA; **f** men with normal-low physical performance and *specific* BIVA; **g** women with normal-low strength and classic BIVA; **h** women with normal-low strength and *specific* BIVA; **i** women with normal-low muscle quantity and classic BIVA; **j** women with normal-low muscle quantity and *specific* BIVA; **k** women with normal-low physical performance and classic BIVA; **l** women with normal-low physical performance and *specific* BIVA; *R/H* resistance standardized for height, *Xc/H* reactance standardized for height, *Rsp* resistance standardized for height and transverse areas, *Xcsp* reactance standardized for height and transverse areas; t^2^: Hotelling’s t-squared statistic

Figure 3 represents the classic and *specific* BIVA for older adults separately grouped according to the sarcopenia criteria (muscle strength [hand grip], muscle quantity [SMI] and physical performance [6MWT]). The mean vectors of groups with low (men = 7; women = 17) and normal muscle strength (men = 22; women = 48) were classified according to their handgrip strength (cutoff: 30 and 20 kg for men and women, respectively) [38]. For Handgrip strength both classic and *specific* BIVA were not significantly different between groups (*p* > 0.05; Fig. 3a, b, g, h). The mean vectors of groups with low (men = 10; women = 12) and normal (men = 19; women = 53) muscle quantity of SMI (cutoff 7.26 kg/m^2^ and 5.45 kg/m^2^ for men and women, respectively) [39] were significantly different between groups in the classic BIVA; and for women in the *specific* BIVA (Fig. 3c, i, j). As expected, the classic and *specific* mean of men and women with low muscle quantity (hollow circle) were positioned in the right region (Fig. 3c, i, j), indicating a smaller amount of body cell mass.

Groups with slow (men = 21; women = 8) and normal (men = 38; women = 27) physical performance were clustered using 6MWT (cutoff < 400 m). The mean vectors of groups were not statistically different for classic and *specific* BIVA (*p* < 0.05) for both sexes in most comparisons (Fig. 3e, f, l), except for women in the classic BIVA (Fig. 3k).

## Discussion

Our findings suggest that classic BIVA presented a distinct association between BC, hydration, physical performance and sarcopenia in older men and women. Data showed that classic BIVA variables were highly associated with two variables: handgrip strength and TBW for men with and without sarcopenia, respectively, whereas it was associated with ALST% and SMI for women with and without sarcopenia, respectively. In *specific* BIVA, the same variable presented the highest correlation coefficients for older adults with and without sarcopenia, for men were TBW%, and for women FM, and FM% for both. In addition, we found that classic and *specific* BIVA were able to distinguish sarcopenia in women, but not in men (Fig. 2). When the sarcopenia criteria were used individually, both classic and *specific* BIVA were able to distinguish muscle reduction (SMI) in women, but only classic BIVA was able to do so for men. For the physical performance criterion, the classic BIVA showed differences only for women (Fig. 3k).

In the classic BIVA, adjustments are made for height to reduce the effect of conductor length [23]. Classic BIVA can be used to detect body fluids and hydration changes and has been proved to be a valid technique for TBW assessment [23, 41, 42], as our results for men without sarcopenia. We found relationships between classic BIVA and hand grip strength in adults with sarcopenia. This association can be explained because the dielectric properties of cell membranes are related to the area and integrity of cell membranes. Integrity of cell membranes is a determinant of membrane potential and, together with area, thereby probably a determinant of cell function [43]. Furthermore, is known that vector migration in the RXc-score graph is associated with an increase in handgrip strength [44]. The *specific* BIVA indicated concordance with the same variable of non-sarcopenic and sarcopenic for men (TBW%) and women (FM), suggesting greater sensitivity of the *specific* BIVA to identify body components. This occurs because the bioelectrical values are also corrected by the cross-sectional areas and this can reduce the effect of body dimensions [45] increasing the sensitivity of bioelectrical values to identify tissues' properties and body composition [46]. Moreover, significant correlation between specific vector and FM% suggests that specific BIVA could be helpful to evaluate sarcopenic obesity diagnosis [47]. Similar associations were also evident in other studies and population groups both at the whole body [22, 45, 46] and segmental level [23]. *Specific* BIVA has been considered adequate as it has been validated against DXA, showing high sensitivity and specificity in the evaluation of %FM [22, 47].

In this study, it was observed that the PA was lower in sarcopenic women, a result that was not replicated among men probably because of the low statistical power of the relatively small sample. In a study with 207 older adults found that sarcopenic individuals had a PA lower than patients without sarcopenia (*p* < 0.05) for both sexes [47]. The PA depends on several biological factors, including the integrity and functionality of the cell membrane, intracellular composition and the ratio of extracellular to intracellular water [48]. A high amount of extracellular water reduces the PA, while a higher proportion of intracellular water is reflected by a higher PA [48].

The classic and *specific* BIVA were sensitive to distinguish sarcopenia in older women (Fig. 2c, d). Although the technique does not imply any direct evaluation of sarcopenia, individuals whose vectors lay to the left of the major axis were characterized by higher body cell mass (without sarcopenia) than those whose vectors were to the right (with sarcopenia). To the best of our knowledge, only one previous research found that both classic and *specific* BIVA were able to identify sarcopenia in older adults [47]. However, they classified sarcopenia using only SMI values (7.26 kg/m^2^ for men and 5.45 kg/m^2^ for women) [39]. In our study, we used three criteria (Handgrip Strength, SMI and 6MWT) [3]. The inclusion of strength and physical performance criteria is important since muscle strength is better than mass in predicting adverse outcomes [2, 49, 50]. Our results indicated that both classic and specific BIVA were not able to distinguish sarcopenia for men. Part of this result can be explained by the different criteria used, and the typical differences in body composition [33], strength levels [51] and hydration [9] between the sexes, as we shown in Table 1, which should directly impact on recent indicators for the classification of sarcopenia (handgrip and SMI). This result should be interpreted with caution given that this study was developed with a specific population of older adults and with small size of the male sarcopenic group. Furthermore, as this issue has been little explored by the scientific literature, there are few comparative results that enable to obtain a more consistent and conclusive information on the use of BIVA as a sarcopenia marker.

Our results (Fig. 3a, b, g, h) showed that BIVA has not enough sensitivity to distinguish strength levels differences from the cutoff points established [38]. On other side, for muscle quantity criterion (SMI), both classic and specific BIVA were able to distinguish sarcopenia in women, and only classic BIVA for men (Fig. 3c, i, j). For the physical performance criterion (6MWT), the classic BIVA showed differences only for women (Fig. 3k). These differences are a well-known expression of the sexual dimorphism of body composition [33]. Women have a cross-sectional muscle area between 25 and 45% smaller than men, lesser amounts of type I fibers, which also gives them less muscle strength than men [51]. From these results, we can infer that as the BIVA indicates variations in tissue hydration and body cell mass, its use to identify sarcopenia is justified in terms of body components of a morphological character. On the other hand, changes in strength (handgrip) seem to depend more on the integrity of the nervous system than on muscle reduction [52]. Thus, this may explain why handgrip and 6MWT were not sensitive to BIVA. Therefore, both BIVAs cannot infer functionality in older adults.

The current investigation has several strengths. As far as we know, this is the first study investigating the association between classic and *specific* BIVA with BC, hydration, and physical performance through DXA and dilution techniques to identify sarcopenia in older adults. From our study, correlation values between classic and *specific* bioelectric variables were generated. Thus, our correlation values can be used in BIVA calculations as reference values for older adults Brazilians with and without sarcopenia. We used the current EWGSOP criterion to identify sarcopenia (by strength as the first criterion), in an actualized way [3]. In addition, for the first time, each criterion was tested individually. Despite the promising results obtained in this study, some limitations are present and should be considered. We used reference values from the Italian population since BIVA values of Brazilian older adults were not yet available. Another point to consider is the sample size and the cross-sectional design of the study which limit the extrapolation of our finds. In addition, the greater participation of women than men can impact the findings. Another limitation is the low number of older adults with sarcopenia (10.6%). However, this value is similar to the worldwide prevalence (10%) [53].

Monitoring the BC and hydration is a relevant topic during ageing mainly because age-related changes in fat, skeletal muscle mass and strength losses are associated with various adverse health outcomes, including a higher risk for disability, morbidity and early mortality [54, 55]. These findings are certainly of interest in clinical practice since many countries around the world are experiencing a change in the age distribution of their populations, with worrying economic impacts [56]. In this sense, BIVA shows to be a promising and inexpensive resource for regular and reliable health monitoring in older adults. Then, the risk diagnosis can be made earlier.

## Conclusion

Our findings demonstrated that the classic BIVA could be used to analyze absolute hydration (TBW) for men without sarcopenia. Equally, classic BIVA monitors variations in muscle index (SMI) and limbs relative muscle mass (ALST%) for women without and with sarcopenia, respectively. Regardless of sarcopenia status, the highest correlation coefficients between *specific* BIVA were observed in the sex-dependent variables of the older adults (TBW% for men and FM for women). Furthermore, the *specific* BIVA could be helpful to assess sarcopenic obesity diagnosis. Both classic and *specific* BIVA were able to distinguish sarcopenia in women.

Both classic and *specific* BIVA were sensitive to individually detect morphological changes, but not the functional criteria of sarcopenia. It is possible to state that BIVA procedures are promising tools to monitor body changes in ageing at the risk of sarcopenia. In the current context of prioritizing functional criteria in the diagnosis of sarcopenia, BIVA shows potential as a confirmatory alternative, economically viable and good sex-dependent morphological sensitivity.

## Data Availability

The datasets generated during and/or analysed during the current study are available from the corresponding author on reasonable request.

## Abbreviations

BMC: Bone mineral content
LST: Lean soft tissue
TBW: Total body water
FM: Fat mass
BC: Body composition
DXA: Dual-energy X-ray absorptiometry
BIA: Bioelectrical impedance analysis
BIVA: Bioelectrical impedance vector analysis
R/H: Resistance standardized for height
Xc/H: Reactance standardized for height
Rsp: Resistance standardized for height and transverse areas
Xcsp: Reactance standardized for height and transverse areas
MMSE: Mini-mental state examination
BMI: Body mass index
SMI: Skeletal muscle mass index
ALST: Appendicular lean soft tissue
6MWT: 6-Minute walk test
R: Resistance
Xc: Reactance

## References

[1] Cruz-Jentoft AJ, Sayer AA (2019). Sarcopenia. Lancet.

[2] Schaap LA, van Schoor NM, Lips P, Visser M (2018). Associations of sarcopenia definitions, and their components, with the incidence of recurrent falling and fractures: the longitudinal aging study Amsterdam. J Gerontol A Biol Sci Med Sci.

[3] Cruz-Jentoft AJ, Bahat G, Bauer J, Boirie Y, Bruyère O, Cederholm T (2018). Sarcopenia: revised European consensus on defnition and diagnosis. Age Ageing.

[4] Malmstrom TK, Miller DK, Simonsick EM, Ferrucci L, Morley JE (2016). SARC-F: a symptom score to predict persons with sarcopenia at risk for poor functional outcomes. J Cachexia Sarcopenia Muscle.

[5] Beaudart C, Biver E, Reginster JY, Rizzoli R, Rolland Y, Bautmans I (2017). Validation of the SarQoL®, a specific health-related quality of life questionnaire for Sarcopenia. J Cachexia Sarcopenia Muscle.

[6] JafariNasabian P, Inglis JE, Reilly W, Kelly OJ, Ilich JZ (2017). Aging human body: changes in bone, muscle and body fat with consequent changes in nutrient intake. J Endocrinol.

[7] St-Onge MP, Gallagher D (2010). Body composition changes with aging: The cause or the result of alterations in metabolic rate and macronutrient oxidation?. Nutrition.

[8] Hooper L, Bunn D, Jimoh FO, Fairweather-Tait SJ (2014). Water-loss dehydration and aging. Mech Ageing Dev.

[9] Serra-Prat M, Lorenzo I, Palomera E, Ramírez S, Yébenes JC (2019). Total body water and intracellular water relationships with muscle strength, frailty and functional performance in an elderly population. J Nutr Health Aging.

[10] Heymsfield SB, Ebbeling CB, Zheng J, Pietrobelli A, Strauss BJ, Silva AM (2015). Multi-component molecular-level body composition reference methods: evolving concepts and future directions. Obes Rev.

[11] Toomey CM, Cremona A, Hughes K, Norton C, Jakeman P (2015). A review of body composition measurement in the assessment of health. Top Clin Nutr.

[12] Kaminsky LA, Ozemek C, Williams KL, Byun W (2014). Precision of total and regional body fat estimates from dual-energy X-ray absorptiometer measurements. J Nutr Health Aging.

[13] Schoeller DA, van Santen E, Peterson DW, Dietz W, Jaspan J, Klein PD (1980). Total body water measurement in humans with 18O and 2H labeled water. Am J Clin Nutr.

[14] Dehghan M, Merchant AT (2008). Is bioelectrical impedance accurate for use in large epidemiological studies?. Nutr J.

[15] Ellis KJ, Wong WW (1998). Human hydrometry: comparison of multifrequency bioelectrical impedance with 2H_2_O and bromine dilution. J Appl Physiol.

[16] Jackson AA, Johnson M, Durkin K, Wootton S (2013). Body composition assessment in nutrition research: value of BIA technology. Eur J Clin Nutr.

[17] Bioelectrical impedance analysis in body composition measurement: National Institutes of Health Technology Assessment Conference Statement. Am J Clin Nutr. 1996;64(3 Suppl):524S–32S. 10.1093/ajcn/64.3.524S. 10.1093/ajcn/64.3.524S 8780375

[18] Kyle UG, Bosaeus I, De Lorenzo AD, Deurenberg P, Elia M, Gómez JM, Heitmann BL, Composition of the ESPEN Working Group (2004). Bioelectrical impedance analysis–part I: review of principles and methods. Clin Nutr.

[19] Piccoli A, Rossi B, Pillon L, Bucciante G (1994). A new method for monitoring body fluid variation by bioimpedance analysis: the RXc graph. Kidney Int.

[20] Norman K, Stobäus N, Pirlich M, Bosy-Westphal A (2012). Bioelectrical phase angle and impedance vector analysis–clinical relevance and applicability of impedance parameters. Clin Nutr.

[21] Buffa R, Mereu E, Comandini O, Ibanez ME, Marini E (2014). Bioelectrical impedance vector analysis (BIVA) for the assessment of two-compartment body composition. Eur J Clin Nutr.

[22] Buffa R, Saragat B, Cabras S, Rinaldi AC, Marini E (2013). Accuracy of specific BIVA for the assessment of body composition in the United States population. PLoS ONE.

[23] Stagi S, Irurtia A, Rosales Rafel J, Cabras S, Buffa R, Carrasco-Marginet M, Castizo-Olier J, Marini E (2021). Segmental body composition estimated by specific BIVA and dual-energy X-ray absorptiometry. Clin Nutr.

[24] Bolfarine H, Bussab WO (2005). Elementos de amostragem.

[25] Visser M, Pahor M, Tylavsky F, Kritchevsky SB, Cauley JA, Newman AB (2003). One- and two-year change in body composition as measured by DXA in a population-based cohort of older men and women. J Appl Physiol.

[26] Icaza MC, Albala C, Projeto SABE (1999). Minimental state examination (MMSE) del estudio de dementia en Chile: análisis estatístico.

[27] Lohman T, Roche A, Martorell R (1988). Anthropometric standardization reference manual.

[28] Venturini ACR, Silva AM, Abdalla PP, Dos Santos AP, Borges FG, Alves TC (2021). Estimating resting energy expenditure from dual-energy X-ray absorptiometry: a cross-sectional study in healthy young adults. Am J Hum Biol.

[29] Abdalla PP, Silva AM, Venturini ACR, Santos APD, Carvalho ADS, Siqueira V (2020). Cut-off points of appendicular lean soft tissue for identifying sarcopenia in older adults in Brazil: a cross-sectional study. Nutr Hosp.

[30] Lukaski HC, Piccoli A, Preedy VR (2012). Bioelectrical impedance vector analysis for assessment of hydration in physiological states and clinical conditions. Handbook of anthropometry.

[31] Piccoli A, Nigrelli S, Caberlotto A, Bottazzo S, Rossi B, Pillon L (1995). Bivariate normal values of the bioelectrical impedance vector in adult and elderly populations. Am J Clin Nutr.

[32] Stagi S, Ibáñez-Zamacona ME, Jelenkovic A, Marini E, Rebato E (2021). Association between self-perceived body image and body composition between the sexes and different age classes. Nutrition.

[33] Saragat B, Buffa R, Mereu E, De Rui M, Coin A, Sergi G (2014). Specific bioelectrical impedance vector reference values for assessing body composition in the Italian elderly. Exp Gerontol.

[34] Cruz-Jentoft AJ, Baeyens JP, Bauer JM, Boirie Y, Cederholm T, Landi F, Martin FC, European Working Group on Sarcopenia in Older People (2010). Sarcopenia: European consensus on definition and diagnosis: report of the European Working Group on sarcopenia in older people. Age Ageing.

[35] Alexandre Tda S, Duarte YA, Santos JL, Wong R, Lebrão ML (2014). Prevalence and associated factors of sarcopenia among elderly in Brazil: findings from the SABE study. J Nutr Health Aging.

[36] Massy-Westropp NM, Gill TK, Taylor AW, Bohannon RW, Hill CL (2011). Hand grip strength: age and gender stratified normative data in a population-based study. BMC Res Notes.

[37] Lourenço RA, Pérez-Zepeda M, Gutiérrez-Robledo L, García-García FJ, Rodríguez Mañas L (2015). Performance of the European Working Group on Sarcopenia in Older People algorithm in screening older adults for muscle mass assessment. Age Ageing.

[38] Lauretani F, Russo CR, Bandinelli S, Bartali B, Cavazzini C, Di Iorio A (2003). Age-associated changes in skeletal muscles and their effect on mobility: an operational diagnosis of sarcopenia. J Appl Physiol.

[39] Baumgartner RN, Koehler KM, Gallagher D, Romero L, Heymsfield SB, Ross RR (1998). Epidemiology of sarcopenia among the elderly in New Mexico. Am J Epidemiol.

[40] Morley JE, Abbatecola AM, Argiles JM, Baracos V, Bauer J, Bhasin S (2011). Sarcopenia with limited mobility: an international consensus. J Am Med Dir Assoc.

[41] Bronhara B, Piccoli A, Pereira JC (2012). Fuzzy linguistic model for bioelectrical impedance vector analysis. Clin Nutr.

[42] Piccoli A (2014). Estimation of fluid volumes in hemodialysis patients: comparing bioimpedance with isotopic and dilution methods. Kidney Int.

[43] Stark G (2005). Functional consequences of oxidative membrane damage. J Membr Biol.

[44] Norman K, Pirlich M, Sorensen J, Christensen P, Kemps M, Schütz T (2009). Bioimpedance vector analysis as a measure of muscle function. Clin Nutr.

[45] Marini E, Sergi G, Succa V, Saragat B, Sarti S, Coin A (2013). Efficacy of specific bioelectrical impedance vector analysis (BIVA) for assessing body composition in the elderly. J Nutr Health Aging.

[46] Marini E, Campa F, Buffa R, Stagi S, Matias CN, Toselli S (2020). Phase angle and bioelectrical impedance vector analysis in the evaluation of body composition in athletes. Clin Nutr.

[47] Marini E, Buffa R, Saragat B, Coin A, Toffanello ED, Berton L (2012). The potential of classic and specific bioelectrical impedance vector analysis for the assessment of sarcopenia and sarcopenic obesity. Clin Interv Aging.

[48] Reljic D, Zarafat D, Jensen B, Herrmann HJ, Neurath MF, Konturek PC (2020). Phase angle and vector analysis from multifrequency segmental bioelectrical impedance analysis: new reference data for older adults. J Physiol Pharmacol.

[49] Ibrahim K, May C, Patel HP, Baxter M, Sayer AA, Roberts H (2016). A feasibility study of implementing grip strength measurement into routine hospital practice (GRImP): study protocol. Pilot Feasibility Stud.

[50] Leong DP, Teo KK, Rangarajan S, Lopez-Jaramillo P, Avezum A, Orlandini A (2015). Prognostic value of grip strength: findings from the Prospective Urban Rural Epidemiology (PURE) study. Lancet.

[51] Miller AE, MacDougall JD, Tarnopolsky MA, Sale DG (1993). Gender differences in strength and muscle fiber characteristics. Eur J Appl Physiol Occup Physiol.

[52] Clark BC (2019). Neuromuscular changes with aging and sarcopenia. J Frailty Aging.

[53] Shafiee G, Keshtkar A, Soltani A, Ahadi Z, Larijani B, Heshmat R (2017). Prevalence of sarcopenia in the world: a systematic review and meta- analysis of general population studies. J Diabetes Metab Disord.

[54] Toss F, Wiklund P, Nordström P, Nordström A (2012). Body composition and mortality risk in later life. Age Ageing.

[55] Santanasto AJ, Goodpaster BH, Kritchevsky SB, Miljkovic I, Satterfield S, Schwartz AV (2017). Body composition remodeling and mortality: the health aging and body composition study. J Gerontol A Biol Sci Med Sci.

[56] Beaudart C, Rizzoli R, Bruyère O, Reginster JY, Biver E (2014). Sarcopenia: burden and challenges for public health. Arch Public Health.

